# Factors influencing recruitment and retention of male nurses in Macau and mainland China: a collaborative, qualitative study

**DOI:** 10.1186/s12912-020-00497-9

**Published:** 2020-11-10

**Authors:** Aimei Mao, Jialin Wang, Yuan Zhang, Pak Leng Cheong, Iat Kio Van, Hon Lon Tam

**Affiliations:** 1 Kiang Wu Nursing College of Macau, Est. Repouso No.35, Macau, China; 2 College of Nursing, Chengdu University of Traditional Chinese Medicine, 1166 Liutai Road, Wenjiang District, Chengdu, Sichuan China; 3 Neijiang Health Vocational College, 368 East Section 4, Han-an Avenue, Dongxing District, Neijiang, Sichuan China

**Keywords:** Male nurses, Professional development, Qualitative research, Mainland, Macau

## Abstract

**Background:**

Macau and the Mainland China have different political and socio-economic policies but are both influenced by Chinese culture. By comparing the professional development experiences of male nurses from Macau and the Mainland, this study aims to explore factors influencing the recruitment and retention of male nurses.

**Methods:**

A collaborative, qualitative approach was adopted in which researchers from Macau and the Mainland were jointly involved in carrying out interviews and analyzing data. A total of 24 clinical male nurses were invited, with 12 each from Macau the Mainland. Recruitment was based on purposive sampling from various health institutions in the two regions. Semi-structured interviews were conducted in 2017–2018 with similar interview guidelines for both Macau and the Mainland sites. Thematic analysis was used for data analysis, and Nvivo11 Plus software was used to facilitate the analysis.

**Results:**

Key facilitators/barriers to recruitment and retention of male nurses were clustered under the two research questions: 1) What are the factors influencing the recruitment of male nurses? 2) What are the factors influencing the retention of male nurses? Males in Macau and the Mainland experienced pressure while entering nursing because of the stereotype that nursing is a feminine occupation. However, males in Macau chose nursing as a college major under their own volition while males in the Mainland were mostly forced into nursing. The males in Macau hardly thought of leaving nursing while their Mainland counterparts constantly felt uncertain about their professional future. The males on both sides hoped to thrive in career development. While the Macanese tended to pursue advanced programs in specialty nursing for better health care in the frontline, the Mainlanders wanted to get promoted to leave the frontline.

**Conclusion:**

Male nurses in Macau and Mainland share some common experiences in professional development but have different views and values regarding nursing.

## Background

The nursing workforce shortage has been a long-term problem faced by both developed and developing countries and regions. The World Health Organization (WHO) predicts a shortfall of nine million nurses worldwide by 2030 [1]. The nurse workforce shortage is likely the single biggest threat to the Universal Health Coverage, a plan endorsed by the United Nations advocating that everyone has access to quality health services without facing financial hardship [2]. As 90% of the nurse workforce worldwide is female [3], attention should be paid to recruiting men into nursing. By hiring more men into the nurse population, the shortage in nursing human resources can potentially be alleviated [4].

Despite the efforts in recruiting men in recent years, nursing is still a female-dominated career. In the United States, a steady increase of male nurses has been observed in the past four decades. However, male nurses remain at 11% of all full-time nurses in the past five years [5]. There are also differences in male nurse proportions in some of the Chinese culture influenced regions of Eastern Asia. In Taiwan, males accounted for 2.06% of the nursing workforce in 2016 [6], while in Hong Kong, 13.4% of the active registered nurses were males in 2016 [7]. More research is needed to explore why gender disparity still exists in nursing and why the discrepancy is more significant in some countries and regions than others. Such exploration would obtain in-depth knowledge of context-related facilitators and barriers influencing men’s decisions to join and stay in nursing.

Previous research has found various barriers men encounter when they choose nursing [6, 8]. Male nurses are often considered to be unsuitable caregivers. Prevailing definitions of masculinity and the questioning of their ability to provide appropriate care have acted as powerful barriers preventing men from entering nursing [9]. Further, men who have joined nursing tend to be confined to specific clinical departments or even be refused services in clinical wards [10]. While some males may cope with these challenges successfully, others struggle, leading to a higher proportion of attrition among male nurses as compared to females [11, 12]. Provided that gender expectations generate a social attitude toward men’s prospect as a nurse, attention should be shifted from personalized aspects, like men’s professional competency and coping skills, to social-cultural restraints that may deter men’s development in the nursing profession.

Cultural norms and socio-economic conditions often shape individuals’ professional identity [13]. It is hinted that males in the socioeconomically disadvantaged background may have different expectations to join nursing from those in affluent conditions. For example, a study of Japanese male nurses in rural areas showed that Japanese men joined nursing mainly because they could not find alternative jobs after economic bubbles broke in the early 1990s when economic deterioration resulted in many companies reducing the number or wages of their employees [14]. In Taiwan, another economically developed region, a substantial proportion of male nursing students enter nursing because of their parents’ choices and economic benefit is an essential consideration [15]. This highlights the family’s role in shaping young people’s future career. Scholars suggest that more studies are needed in different regions of varying economic conditions to obtain an in-depth knowledge of the facilitators of and hinderances to men joining and staying in nursing [14].

The purpose of this study is to explore the factors influencing the recruitment and retention of male nurses in Macau and the Mainland of China (Mainland). It will answer two questions: 1) What are the factors influencing the recruitment of male nurses? 2) What are the factors influencing the retention of male nurses? A combination of the data from the two regions with similar cultures but different socio-economic contexts can enrich the findings on male nurses’ professional development.

## Methods

### The design

This study is a collaborative, qualitative research approach in which researchers from Macau and the Mainland were jointly involved in carrying out interviews and analyzing data. A collaboration agreement was signed. It was required that the researchers in each of the two regions work under the same research framework at a similar schedule.

### Participants

The eligibility criteria for participants were: 1) male registered nurses; 2) frontline nursing staff with direct patient contact; 3) at least one-year work experience. Purposive sampling, supplemented by snowballing sampling, was used to recruit the participants. Different backgrounds of the participants were considered to maximize a heterogeneous sample, such as the health institutions or departments employed, the length of time working as a nurse, educational level in nursing, marital status, and professional positions, etc.

The participants on the Macau side came from both public and private health institutions. Macau is one of the two Special Administrative Regions of China, along with Hong Kong. It is now under a “one country, two systems” political policy and thus enjoys much more autonomy than the Mainland of China. Macau is a developed region with a Gross Domestic Product (GDP) per capita of USD 86000 in 2018 [16], making it one of the richest regions in the world. Mainland has witnessed dramatic economic development in the past four decades. However, with USD 9770 per capita GDP [16], it is still considered a developing country. Macau currently has 270 male registered nurses, accounting for 11% of the nursing workforce, compared to 2.1% in Mainland [17, 18]. In Macau, the majority of its citizens are Chinese descendants, implying that it is a society influenced by Chinese values and cultures.

The participants on the Mainland side were recruited from six tertiary hospitals, the highest-ranking hospitals, from Chengdu, Sichuan Province in Western China. Chengdu is the capital city of Sichuan Province, with a population of 16,330,000. Due to their small number, male nurses in China always enjoy advantages in the job market and most of them get employment in the tertiary hospitals.

All the researchers were registered nurses and faculty members of the nursing colleges in the local regions. Initially, they recruited the participants by their connections with the potential participants or by previous participants’ recommendations of new participants. As the pool of potential participants quickly expanded, the researchers selected those who might add more valuable information than others. An interview appointment was made once the potential participant expressed willingness to take part in the study. The two regions conducted the interviews concurrently, and their interviews were combined and analysed as a whole. A total of 24 clinical male nurses were invited, with 12 from Macau and the other 12 from the Mainland.

### Data collection

Semi-structured interviews were used to collect data. A similar interview guide was used by the researchers from the two sides (Table 1). The interview questions were developed by the researchers based on their literature review and their experiences as clinical nurses and nurse educators.

**Table 1:** The interview guideline

The open-ended questions	
1. What do you think about nursing?	
2. How did you join nursing? Please tell me the story of how you joined nursing.	
3. How did your family or friends react when you decided to join nursing?	
4. What advantages have you come across as a male nurse? What are the disadvantages?	
5. What is your plan in the future?	

The interviews were conducted at a convenient time and place for the participants and recorded under their permission. One-to-one interviews were conducted with one researcher (interviewer) and one participant (interviewee). Probing was used to clarify the vague points and dig deep into the participants’ underlying values and attitudes related to nursing. The average time was 50 min for each interview in Macau and 58 min in the Mainland. About 15 min were taken before interviewing for the interviewers to explain the study and for the interviewees to sign the consent form. Field notes were written immediately after each interview. As the interviews from the two sides accumulated, repeated information indicated data saturation, pointing to termination of further interviews.

### Data analysis

Recorded interviews were transcribed verbatim by a transcription business agent. A contract was signed to ensure the confidentiality of the data and immediate destruction of the audio recordings of the interviews as soon as the transcriptions were finished. A thematic analytic process was used, following the four steps [19]. Firstly, the researchers read the transcripts several times, sometimes against the recordings, to get familiar with data; secondly the researchers looked for meaningful data sets, and assigned the sets with codes; thirdly, the researchers identified the relationships between the codes. By comparing the data from Macau and Mainland, data were interrogated for similarities and differences in the experiences of the male nurses in the two regions. The researchers then grouped the related codes into sub-themes and sub-themes into themes; fourthly, the researchers formulated the themes as the expression of the latent of the transcribed interviews. The qualitative data analysis software, Nvivo11 Plus, was used to facilitate analytic processing. The Macanese team, who had more qualitative research experiences than the team members in the mainland, established the analytic framework and led the data analysis. The researchers from the mainland were also sufficiently involved in the data analysis and the two teams frequently discussed data analysis via WeChat, a popular social media in China and Macau. The final analytic results were approved by both sides.

### Ethical consideration

Each research team received approvals from the research committee of their respective colleges. The study in Mainland was approved as a postgraduate candidate’s degree-fulfillment program. In Macau, the study was part of a larger research project, which was funded by Foundation of Macau (No:1962/DSDSC/2016). Each participant was provided an information sheet with an assurance that their information would be kept confidential and they had the right to withdraw from the study at any time.

### Rigor

The research rigor was established by using the four criteria advocated by Lincoln & Guba [20]: credibility, dependability, confirmability, and transferability. Credibility is to build confidence that the results of the study are true and believable. This study achieved credibility through the researchers’ authority and member check. The study was conducted by a group of researchers who had all been trained in qualitative research and who were registered nurses. One of the researchers was a male nurse who had worked as a frontline nurse for several years. The similarity of professional background between the researchers and those under scrutiny had helped the processes in interviewing and data analysis. The researchers conducted member checks by sending the analytic results back to several of the participants to obtain their feedback. Dependability means that the study findings are repeatable if the study occurs within the same participants, researchers and settings. A rich description of the study methods was offered in this study to enhance dependability. The collaborative research design was also proof of dependability as the researchers in both Macau and Mainland conducted the research in the same way and got comparable results. Confirmability can extend the confidence that other researchers would confirm the study results. The researchers on the two sides regularly corresponded during data collection and data analysis to get consensus. Transferability means that study results can be generalized to other contexts or settings. The collaborative research design enhanced transferability because this research approach ensured a wide range of participants. Also, the purposive sampling in the study maximized the sample diversity, which increased the transferability of the study findings.

## Results

### Main findings

The researchers recruited 24 participants, with each of the two regions having 12. The characteristics of the participants are provided in Table 2. A relatively clear path of professional development can be captured from the male nurses, beginning from their college major choice to current career. The participants from both sides experienced a fluctuation in their career development, particularly in the first years of their career. After surviving the transition to professional life, they felt settled down and considered long-term career development. Despite challenges, participants from Macau rarely planned to leave nursing, while the Mainlanders often contemplated leaving.

**Table 2:** Characteristics of the male nurses from Macau and Mainland (*N* = 24)

Characteristics	Males from Macau (12)	Males from Mainland (12)	Total
Age-median and range (year)	27.2 (23–33)	28.7 (23–33)	27.9 (23–33)
Years of employment-median and range	5.2 (1–11)	5.4 (1.5–10)	5.3 (1–11)
Marital status			
Single	7 (58.3%)	6 (50.0%)	13 (54.2%)
Married	5 (41.7%)	5 (41.7%)	10 (41.7%)
Divorce	0 (0%)	1 (8.3%)	1 (4.1%)
^a^Current employment			
ER	5 (41.6%)	2 (16.7%)	7 (29.2%)
ICU	2 (16.7%)	5 (41.6%)	7 (29.2%)
HU	2 (16.7%)	0 (0%)	2 (8.3%)
OR	0 (0%)	3 (25.0%)	3 (12.5%)
Other	3 (25.0%)	2 (16.7%)	5 (20.8%)
Positions			
Head-nurse (or nurse specialist)	1 (8.3%)	1 (8.3%)	2 (8.3%)
Group-head	1 (8.3%)	4 (33.3%)	5 (20.8%)
Staff nurse	10 (83.4%)	7 (58.4%)	17 (70.9%)

^a^*ER* emergency room, *ICU* Intensive Care Unit, *HU* Hemodialysis Unit, *OR* Operation Room

**Fig. 1:**
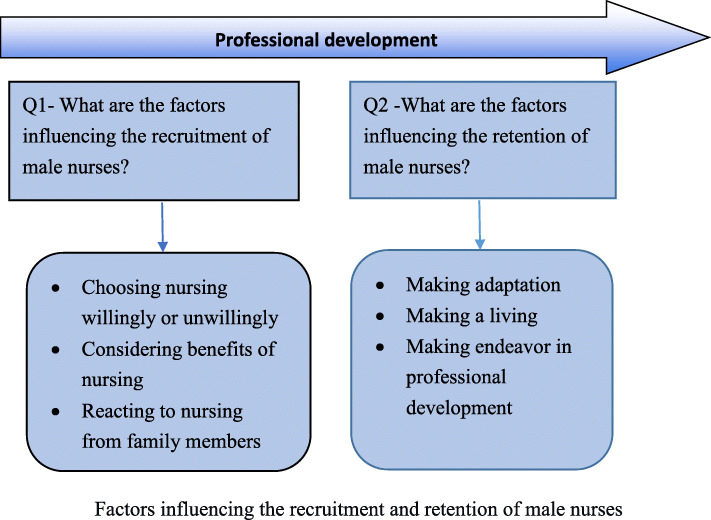
Factors influencing the recruitment and retention of male nurses

Macanese participants tended to seek specialty nursing. The Mainlanders found it challenging to find jobs outside of nursing when trying to leave. As a consequence, the Mainlanders sought opportunities to leave frontline nursing instead of leaving nursing completely. Several themes emerged under each of the two research questions: Q1- What are the factors influencing the recruitment of male nurses? Q2 -What are the factors influencing the retention of male nurses? (Shown in Fig. 1).

In the following sections, descriptions of the themes are supported by extracts from the interviews. Participants were coded to conceal their identity, with Macau male nurses coded as Mmn and Mainland China male nurses as Cmn.

### Q1- what are the factors influencing the recruitment of male nurses?

Choice of college and major was one of the most critical decisions made by the participants. All participants, some of whom had worked for more than ten years, vividly described how they made a choice. While some of them made their choice carefully and knowingly, most made a choice contingently.

#### Choosing nursing willingly or unwillingly

In Macau, there is no regional-wide college entrance examination, so all Macanese can make their own decision on college majors. Seven of the 12 participants made nursing their first major choice, while the others had nursing as one of several options.

*I put down many universities in my college choice list. But I had nursing as my major in each of the universities. I also chose the two nursing schools in Macau. I had no interest in other majors*. Mmn4.

Macanese participants primarily decided to join nursing schools because they liked to help others, or they perceived that their personality was suited to nursing.

*When I decided on the college major, I wanted a major with the skills that could be useful after graduation. This major should not be like accounting, which I thought a dull career. I like to work with people and nursing is just such a job.* Mmn12.

Among the 12 Mainlanders, only one made nursing his first major choice. Three made nursing one of their choices but recounted that they did not really want to go to nursing. Eight of them did not put nursing as their college choice; they were transferred (tiao-pei) to nursing by college admission officials based on college entrance scores. Cmn5 described that he was forced to join nursing, a typical phenomenon in the university he joined:

*I chose XX University, not this university. So, I was transferred not only to the major but also to the university … I decided to settle down in the university at first, then decide what to do. I had heard the university students could change their major. But I did not realize that it was complicated to change. Everyone in our nursing school seemed to have been transferred to nursing.* Cmn5.

#### Considering the benefits of nursing

All participants mentioned specific benefits as incentives prompting them to pursue nursing. The supposed benefits included the relatively decent salary, the stability of employment and the help with their family members’ health care. The Macanese described their family’s condition and expressed the importance of economic stability for their family.

*(When I chose college major) I did not want to leave Macau. But there were no medical schools in Macau. XX (a teacher in a nursing school in Macau) came to our high school and told us that nursing students had a bright future. They could earn a lot. At that time, my family [economic situation] was difficult. So, I chose nursing without hesitation.* Mmn7.

The economic benefits of nursing were paramount for the Mainlanders. Ten were from the countryside and the other two from small towns. As a minority among nursing professionals, males were told that it would be easy to find a job in big hospitals in big cities. Also, they could provide help once their family members in the countryside fell ill.

*I did choose nursing when I chose a college major. All the majors I chose were medical-related. I came from the countryside. It was difficult for the countryside people to get medical services. I thought that if I would work in a hospital in a city, I could help my family and relatives when they got ill.* Cmn6.

#### Reacting to nursing from family members

The Macanese mentioned that their parents showed mixed feelings for their choice of nursing. Some of their family members and friends were surprised at their choice because few men chose nursing.

*My parents were surprised when they learned that I had chosen nursing as my college major, “You are a boy. Why nursing? Why not police or other careers?”, they asked me.* Mmn1.

Mixed responses existed among the parents of the Mainlanders when they heard of their son’s choice. Some parents showed no support nor opposition because they knew nothing at all about nursing. Some were shocked. Others were somehow happy because their son got accepted by a university and would leave the countryside forever. While some of the families proudly spread their son’s being accepted by a university in the neighborhood, they kept the major as a secret to avoid curiosity and questioning.

*My parents said nothing when they learned that I was transferred to a nursing school. They had no reaction* (sigh). *You know, they are peasants. They knew nothing about nursing.* Cmn12.

*Neighbors would ask what major I was learning at university. My parents would tell them I was studying in XX medical university. You know, they felt challenged to reveal my major. Male nurses were not heard of.* Cmn4.

### Q2 -what are the factors influencing the retention of male nurses?

Participants detailed that it was after they had worked independently as a clinical nurse that they realized what nursing meant. They experienced struggles during transitional times as a neophyte. Once they came out of the transition, they felt settled down and began to think of their future. The participants on both sides sought career advancement, but with different expectations.

#### Making adaptation

The participants on the two sides shared some common challenges during their transition time, such as being unfairly treated by colleagues, being isolated, finding it difficult to independently carry out duties, etc. Three men on the Macau side changed their wards or hospital but still worked as a nurse in their new position.

*There were male nursing students who quit the study because they felt nursing did not match their personality. Once they graduated, very very few would leave nursing. I heard that several male nurses changed their wards or hospitals. But they were still in nursing.* Mmn 9.

Most of the Mainlanders had intended to join nursing temporarily. Some of them had tried other jobs before they came to a hospital as a nurse. However, their own experiences, in addition to their contact with other men who quit nursing, made them realize that the chances to find another decent job was meager. Therefore, they stopped looking for other jobs and began to settle down in nursing. The psychological adaptation from uncertainty to acceptance to nursing career, although being reluctant, was reflected in Cmn1’s descriptions on the destinations of his fellow male schoolmates:

*There were four male nursing students in my class. One quit school because he hated nursing. One became a clinical nurse after graduation but left Sichuan after one year and went to Guangzhou, still in nursing. He earns much more in Guangzhou than in Sichuan. The third did not intend to do nursing from the very beginning when he came to nursing school. He graduated but did not do nursing. He and another quitter, who was a one-year senior to us, developed their own business. Their business failed. He then found a job in a nursing home near his hometown. Two nursing boys in the next-door to our class did not do nursing. They attended the national test for civil servants. They succeeded and joined the government. The other boys who graduated the same year as I did from our nursing school all went to hospitals and became nurses …*. A*bout two-thirds of the male nursing students finally became nurses.* Cmn1.

#### Making a living

All the participants mentioned money issues without prompting. The Macanese expressed satisfaction with their salary, which was much higher than their high school classmates in other jobs.

*Nursing is a hard job. But right now, nurses’ pay is satisfying. You know the salary’ levels in Macau, right? Our salary is much higher than the average. If you leave nursing, you may not be able to find another job with such a salary*. Mmn 11.

The Mainlanders were unsatisfied with their salaries. They were paid much less than other health professionals in their hospital. On the other hand, they expressed that by doing nursing, they could be able to live in a big city and build their own family.

*I would not say that I love nursing, but I should be grateful for this hospital. It provides me a job. As for the salary, it is lower than some people but higher than other people. I have bought my house. I have much pressure to pay the mortgage, with the current salary. But I am satisfied that I live in this big city.* Cmn2.

#### Making endeavor in professional development

All participants began to make a plan for their long-term future after the transition period. The majority of Macanese males hoped to develop into nursing specialists. Nine of the participants were planning or undergoing on-the-job postgraduate programs.

*In our department, about 70–80% of male nurses have attended or are attending advanced nursing programs. As long as males join nursing, they should prepare to develop further. Even if they themselves don’t have the desire, the heads of the ward or department would expect them to do.* Mmn 11.

The Mainlanders were eager to advance their career, too. They were more likely to pursue a promotion in management positions for two benefits: economic gain and freedom from frontline patient care.

*I am now the head of a nursing group in the ward. But I still want to move up a little further, you know what I mean. I want a position free from nightshifts. I am feeling physically unable to bear nightshifts as I am getting older.* Cmn4.

Several males even set a timeframe to get promoted. They would leave the current position or even nursing if they did not achieve their goal within the timeframe, such as ten years of clinical work.

### Discussion

Similarities and differences were found among the male nurses in Mainland China and Macau regarding joining and remaining in nursing. Our study supports previous findings that motivations to induce males into nursing are related to social attitudes toward male nurses and the economic prospects of being nurses [21, 22]. We found that male nurses in Macau and the Mainland were not widely accepted by the public, particularly in the Mainland. It was also hinted from both sides that entry requirements for nursing schools were lower than other majors. Studies in other places have the same findings that high school students had to choose nursing due to their poorer academic performances than others [15, 23]. When high school graduates are competing to enter other health-related majors, such as dentistry, medicine, pharmacy, etc., the “dumping course” of nursing is increasingly devalued by the high school students as well as the public [23]. The low entry requirements may further contribute to the negative social attitudes toward nursing and exert pressure on those college applicants who consider nursing as a major.

Altruism is a motivation for high school graduates to join nursing [23, 24]. However, we found only a small part of the males joined nursing with a passion for helping others. The males of both sides, particularly the Mainland males, entered nursing out of economic consideration. Both sides revealed the financial constraints of male nurses’ families, echoing other studies which suggest that male nurses might be from families of low-middle to low economic conditions [21, 25]. Driven into nursing almost exclusively out of economic benefits, male nurses in China often face a dilemma--while nursing can secure a stable urban life for men coming from the hardships of the countryside and small towns, these men are increasingly vulnerable to leaving nursing when exposed to more job opportunities. This phenomenon, revealed in our study, is also supported by other studies in China [11, 12].

Whereas the Macanese in our present study were committed to nursing and seemed to have a clear path for their career future, the Mainlanders were uncertain about their future. Survey studies in China have revealed generally low levels of commitment to nursing among nurses and even lower commitment among male nurses [26]. Hence, our findings add evidence for the underlying reasons for which Mainland male nurses may have low morale.

Both Macanese and Mainlanders in our study were more likely to work in wards requiring physical strength or specialty nursing, such as ICU, ER, OR, HR, etc. Other studies have uncovered that male nurses prefer to develop expertise in specialty nursing to obtain a dominant position in their group [27–29]. This is possibly a way to demonstrate masculinity. The same supposition was observed in our study that some males endeavored to advance their professional prowess, even when they did not really like nursing. The contradiction between their behaviors and attitudes toward nursing implies the complexity in professional identity, which incorporates the feelings, behaviors, and values of nurses to their profession [30].

### The implication for nursing education and nursing management

Our study suggests that the general Chinese society still seems conservative, to different degrees in different regions, to men’s role as a nurse. As such, nursing schools and health institutions should disseminate more information on the roles of male nurses to establish a positive social image of male nurses. Such dissemination can help increase the recruitment of males into nursing. For example, Queen’s University Belfast carried out a campaign to recruit more male nursing students that included targeting all-boys schools and the university saw a rise of male students from 6 to 10% in three years [31]. It has been suggested that individuals begin to form a professional identity well before joining formal professional training [32]. This is reflected in our study through the finding that some of the Macanese participants had made preparations for their future careers by engaging in health care activities during high school. Therefore, dissemination of information on the roles of nurses could extend from high schools to primary schools to have even more males consider nursing from an earlier age.

Further, despite a shortage of nurses, it would be imprudent to relax entry requirements for the nursing major in a bid to attract young people to join nursing. Relaxing such requirements can, in the long run, be detrimental to the social image of nurses [33]. Rather, as in the case of Macau, subsidies and scholarships from the government and private sectors have been established to attract talented high school graduates to join nursing schools. Given the breathtaking economic achievements in the past decades, the Mainland can provide similar subsidies and scholarships as incentives to attract talented students into nursing.

College admission officials in Mainland should rethink the student enrolment mechanism. In the current situation, nursing is not a popular major valued by college applicants in China and many of the nursing students are transferred to nursing by admission officials after do not obtain sufficient achievement scores for their intended majors during college applications. Numerous studies have revealed a higher level of commitment among those who willingly join nursing than those who feel forced to [34–36]. The utterly different admission mechanisms for nursing students between Macau and Mainland China may have contributed to commitment differences. More importantly, our present study found that the transfers disproportionally affected the students from disadvantaged regions of China because the current admission quota system in China favors the students in large cities, like Beijing and Shanghai. There have been debates in China on equal education rights [37, 38]. Such debate is worth to continue. Indeed, eliminating inequality in the admission of nursing students can create a more committed nurse cohort.

Nursing is not an attractive career from the Macanese and Mainland perspectives. Complaints about low payment were heard from the Mainland male nurses. Thus, nursing scholars and managers in Mainland should advocate for nurses’ interests. Experiences from Macau can be learned to raise nurses’ wages to well above the average level of the society. Further, in an affluent society like Macau, a fair payment system between nurses and other health professionals must be established to increase the attractiveness of nursing. Scholars have pointed out the top ten nursing trends for 2020 and “Increased Specialization” tops the ranks [39]. Our study found this trend among Macanese male nurses but not Mainlanders. Barriers preventing Mainlanders from pursuing specialty nursing need to be lifted. On-the-job postgraduate programs for clinical nurses in the Mainland should be considered, similar to programs in Macau.

### Limitations of the study

Although efforts were made by the researchers to enhance the rigor of the study, this qualitative study has inherent limitations with qualitative research design. The findings represent the experiences and views of a small number of male nurses in Macau and one city in Mainland China. If the males had been recruited from other sources, their views and experiences might have been different. Also, purposive sampling, known as judgmental sampling, is subject to researchers’ bias [40]. Whereas the researchers in the study had carefully established the sampling criteria, it was possible that the criteria did not accurately capture the influencing factors to men’s joining in nursing and better informed participants could have been missed.

## Conclusion

The WHO has named the year 2020 “Year of the Nurse and Midwife” and WHO chief nursing officer has advocated nurse recruitment drives in 2020 [41]. Males will inevitably be a target within the recruitment drive. More studies on incentives to induce men into nursing are needed to increase the recruitment of male nursing students and male nurses. Our study has found shared challenges and coping strategies among male nurses in Macau and Mainland in their career. However, the differences are startling in terms of prospective nurses’ motivations to join nursing and current nurses’ expectations of career advancement. It is expected that our study findings will provide nursing educators and policymakers with considerations for future strategies to enhance nurse recruitment and reduce attrition of the nursing workforce. More collaboration is needed among nursing educators, researchers, and managers to combat the common problems facing nursing in different socio-economic contexts.

## Data Availability

The datasets used and/or analyzed during the current study are available from the first author and/or the corresponding author based on reasonable request.

## Abbreviations

WHO: World Health Organization
ER: Emergency room
ICU: Intensive Care Unit
HU: Hemodialysis Unit
OR: Operation Room
Mmn: Macau male nurse
Cmn: Mainland China male nurse
